# Anhedonia and depression severity dissociated by dmPFC resting-state functional connectivity in adolescents

**DOI:** 10.1177/0269881118799935

**Published:** 2018-09-27

**Authors:** Ewelina Rzepa, Ciara McCabe

**Affiliations:** School of Psychology and Clinical Language Sciences, University of Reading, UK

**Keywords:** Depression, biomarker, resting-state, dmPFC, adolescent, DMN

## Abstract

**Introduction:**

Given the heterogeneity within depression, in this study we aim to examine how resting-state functional connectivity (RSFC) in adolescents is related to anhedonia and depression severity on a continuum in line with the research domain criteria (RDoC) approach.

**Methods:**

We examined how RSFC in the dorsal medial prefrontal cortex (dmPFC), nucleus accumbens (NAcc) and pregenual anterior cingulate cortex (pgACC) was related to anhedonia and depression severity in 86 adolescents (13–21 years).

**Results:**

We found both anhedonia and depression severity related to decreased dmPFC RSFC with the precuneus, a part of the default mode network. However we also found that increased dmPFC connectivity with the ACC/paracingulate gyrus related to anhedonia whereas increased RSFC with the frontal pole related to depression severity.

**Discussion:**

This work extends the view that the dmPFC is a potential therapeutic target for depression in two ways: 1. We report dmPFC connectivity in adolescents; and 2. We show different dmPFC RSFC specific to anhedonia and depression severity, providing neural targets for intervention in young people at risk of depression.

## Introduction

Major depressive disorder (MDD) is estimated to have a lifetime prevalence of approximately 16%, with around 8% of adolescents being affected by depression by the age of 16 years (Saluja et al., 2004). As recognition of MDD in adolescents has increased in recent years, more attention is directed to the aetiology and the consequences of early onset depression. Further, it has been suggested that examining clinical symptoms as a continuum across symptom severity ranges may be more useful for identifying neurobiological signatures and risk markers (Insel et al., 2010).

Resting-state functional connectivity (RSFC) studies have revealed that individuals with depressive symptoms have abnormalities in key RSFC networks such as the salience network (SN) (Manoliu et al., 2014a; van Tol et al., 2013), the central executive network (CEN) (van Tol et al., 2013) and the default mode network (DMN) (Bluhm et al., 2009; Greicius et al., 2007; Manoliu et al., 2014a; Northoff, 2016; Sheline et al., 2010).

The SN, which consists of regions such as the pregenual anterior cingulate (pgACC), the insula and the amygdala, responds to salient stimuli, whereas the CEN, which involves regions such as the dorsolateral prefrontal cortex (dlPFC), dorsal medial prefrontal cortex (dmPFC) and the posterior parietal cortex, is involved in cognition for e.g. attention and working memory (Bressler and Menon, 2010). It has been suggested that the dysfunction in these networks in patients with MDD may be due to poor attentional control over emotional stimuli. Studies have found both increased and decreased SN and CEN RSFC in depression (Horn et al., 2010; Manoliu et al., 2014b; Ramasubbu et al., 2014; Sheline et al., 2010; Tahmasian et al., 2013; Ye et al., 2012). Consistent with this, a recent meta-analysis finds that in first-episode, drug-naïve MDD patients, RSFC alterations were located mainly in the fronto-limbic system, including the dorsolateral prefrontal cortex and putamen, and in the DMN, namely the precuneus and superior/middle temporal gyrus (Zhong et al., 2016). The authors concluded that, as the fronto-limbic circuit and the DMN were each functionally altered, these two networks may contribute, respectively, to emotional dysregulation and maladaptive cognitive patterns (Zhong et al., 2016).

Although few studies have examined RSFC in adolescents with depression, one study reports both increased RSFC between the amygdala and the precuneus and decreased connectivity between the SN and the amygdala and the hippocampus and brain stem, which also correlates with depression severity (Cullen et al., 2014). While Pannekoek et al. (2014) found both increased RSFC between the amygdala and the parietal cortex in MDD adolescents and decreased RSFC between the amygdala and regions such as the pgACC, frontal pole and the paracingulate gyrus, Clasen et al. (2014) also report that adolescents at familial risk of depression have decreased RSFC between the prefrontal cortex and parts of the CEN, which also correlated with the parent’s depression severity. Further Gabbay et al., 2013 found that functional connectivity between the striatum and midline structures, including the precuneus, posterior cingulate cortex, and dmPFC, correlated with MDD severity in 21 adolescents. However, distinct striatal RSFC patterns involving the pregenual ACC, subgenual ACC, supplementary motor area, and supramarginal gyrus, were associated with anhedonia severity. Taken together, it has been suggested that an increase in vulnerability to depression may thus be underpinned by altered development in resting state networks in young people at risk. However this needs to be examined thoroughly using longitudinal designs. Recently, we investigated a group at risk for depression, i.e. adolescents with depressive symptoms but no clinical diagnosis, and we also found decreased RSFC in key networks such as the SN the CEN and the DMN compared with healthy controls (Rzepa and McCabe, 2016), albeit with a small sample size in this study.

As it has been suggested that traditional diagnostic boundaries are not entirely useful for capturing the fundamental underlying mechanisms of psychiatric dysfunction (Insel et al., 2010), our aim in this study was to examine, using a dimensional approach, how RSFC relates to symptoms like anhedonia and depression severity in a much larger sample of adolescents, in line with the research domain criteria (RDoC) approach. Consistent with this, a recent RSFC study examined a range of symptoms in patients with anxiety disorder and MDD and found that adding a dimensional approach to categorical provided a more complete mapping of psychopathology to neurobiology (Oathes et al., 2015).

Based on the previous literature, we selected seed regions that have been shown dysfunctional in depressed patients and in adolescents at increased risk of depression in resting state: specifically, we selected seed regions based on Sheline et al. (2010), which focused on the dorsal nexus (dmPFC) as a key region/hub involved in dysfunctional RSFC in depression in adults (Sheline et al., 2010). As we are interested in RSFC and how it might relate to the symptoms of low mood and anhedonia, we also selected the nucleus accumbens seed as it is a key region involved in the salience network and reward processing. We also selected the pgACC seed from our recent study that found reduced pgACC activity during reward anticipation correlated with anhedonia in young people with depression symptoms (Rzepa et al., 2016a).

## Materials and methods

### Participants

We recruited from the general population adolescents (*n* = 86, aged 13–21 years, M = 18.09, SD = 1.89) (Sawyer et al., 2018) with a range of depression symptoms in line with the RDoC approach. We did this by placing different adverts: an advert for young people with symptoms of depression and an advert for young people with no explicit mention of depression symptoms. Some participants had a depression diagnosis from their GP, a psychologist or a psychiatrist (*n* = 27), some were on antidepressants (*n* = 14) or had a history of antidepressants (*n* = 6) (see Table S3). Therefore the adolescents recruited had a range of depression symptoms as can be seen from the Beck Depression Inventory (BDI) (Table 1). We also combined data from adolescents (*n* = 16) who had high depression symptoms (high BDI and high Mood and Feelings Questionnaire (MFQ) designed for younger participants) from our previous paper (First et al., 1997)). We used the Structured Clinical Interview for DSM-IV Axis I Disorders Schedule (SCID) to exclude for any other psychiatric history (Rzepa et al., 2016a). We excluded left-handed, pregnancy, any contraindications to MRI and any medications except for the contraceptive pill. The National and University Research Ethics Committees approved the study and written informed consent was obtained.

**Table 1.** Demographics.

Measure	Depression symptoms (*n* = 44) Mean (SD)	Healthy controls (*n* = 42) Mean (SD)	p-value
Age (years)	18.11 (1.84)	18.02 (1.94)	.827
Gender	F34, M10	F32, M10	.907
BMI	21.73 (2.24)	21.09 (2.41)	.205
BDI	29.70 (12.69)	3.30 (4.1)	<.001
FCPS	117.23 (25)	137.01 (19.18)	<.001
SHAPS	30.8 (7.34)	21.21 (8)	<.001
TEPS-A	36.25 (8.67)	48 (5.78)	<.001
TEPS-C	30.61 (6.41)	36.76 (7)	<.001

BDI: Beck Depression Inventory; BMI: body mass index; F: females; M: males; FCPS: Fawcett-Clarke Pleasure Scale; SHAPS: Snaith–Hamilton Pleasure Scale; TEPS-A: Temporal Experience of Pleasure Scale, anticipatory subscale; TEPS-C: Temporal Experience of Pleasure Scale, consummatory subscale.

### Depression and anhedonia questionnaires

The MFQ measures depression symptoms in adolescents. Scores on the short version of the MFQ range from 0 to 26, while scores on the long version range from 0 to 66. Higher scores on the MFQ suggest more severe depressive symptoms. Scoring a 12 or higher on the short version and a 27 or higher on the long version may indicate the presence of depression in the respondent. There are no prescribed cut-points for any version the MFQ since there is no single cut-point that is best for use in all circumstances. The BDI measures the severity of depression, from lack of depression to extreme clinical depression. On both of these scales greater depression severity = greater score. The Temporal Experience of Pleasure Scale (TEPS) designed to measure individual trait dispositions in both anticipatory and consummatory experiences of pleasure. High scores = high anticipatory and consummatory pleasure. The Fawcett-Clark Pleasure Scale (FCPS) measures how participants rate imagined hedonic reactions to hypothetical pleasurable situations in the moment, low score = low hedonic capacity (Fawcett et al., 1983). The Snaith-Hamilton Pleasure Scale (SHAPS) measures four domains of state hedonic experience: interest/pastimes, social interaction, sensory experience, and food/drink. Higher SHAPS total scores indicate greater pleasure capacity (Snaith, 1995).

### Overall design

The resting-state data were acquired before any other scans including the structural scan. Subjects were instructed to lie in dimmed light with their eyes open, think of nothing in particular, and not to fall asleep, similar to our previous studies (Cowdrey et al., 2012; McCabe and Mishor, 2011; McCabe et al., 2011; Rzepa et al., 2016b), and a method found to have higher reliability than eyes closed (Patriat et al., 2013).

### Image acquisition

A Siemens Magnetom Trio 3T whole body MRI scanner and a 32-channel head coil were used. Multi-band accelerated echo planar imaging sequencing (Center for Magnetic Resonance Research, Minneapolis, MN) was used with an acceleration factor of 6 and iPAT acceleration factor of 2. T2*-weighted EPI slices were obtained every 0.7 s (TR = 0.7, TE = 0.03). Fifty-four transverse slices with in-plane resolution of 2.4 × 2.4 mm were attained and slice thickness was 2.4 mm. The matrix size was 96 × 96 and the field of view (FOV) were 230 × 230mm. Acquisition was performed during resting-state scan, yielding 420 volumes in total. Sagittal 3D MPRAGE images were also acquired 1 × 1 × 1 (TI = 0.9 s, TR=2.02, flip angle 9°, FOV = 250 × 250 mm).

### fMRI data analysis

#### Pre-processing

fMRI data pre-processing was carried out using FEAT (FMRI Expert Analysis Tool, Version 6.0, a part of FSL software), and included the following steps: non-brain removal (Smith, 2002), motion correction using MCFLIRT (Jenkinson and Smith, 2002), spatial smoothing using a Gaussian kernel of full-width at half maximum (FWHM) of 5 mm, grand mean intensity normalization of the entire 4D dataset by a single multiplicative factor and high pass temporal filtering (Gaussian-weighted least-squares straight line fitting, with sigma = 64.0s). fMRI volumes were registered to the individual’s structural scan and the MNI-152 standard space image (Montreal Neurological Institute, Montreal, QC, Canada) using FMRIB’s Linear Image Registration Tool (FLIRT) (Jenkinson and Smith, 2002).

#### Time series extraction and higher level analysis

To study resting-state functional connectivity, a seed-based correlation approach was used. Using the Harvard-Oxford subcortical structural atlas (Kennedy et al., 1998) we created bilateral nucleus accumbens seeds as these are small structures and are not suitable for a region of interest (ROI) sphere. To maximize the exact coverage, the masks of these seed regions were threshold by 20% to include voxels having at least 80% of probability of being in these particular regions. We also created seeds for the dmPFC (18 34 29; –24 35 28) (6 mm sphere so as to not cross into other brain regions) coordinates from Sheline et al., (2010) and pgACC (8 mm sphere with a centre at 0 38 0 so as to not cross into other brain regions). The dmPFC and pgACC seeds were created with Wake Forest University Pickatlas tool in SPM8 as in our previous study (McCabe et al., 2011).

The mean time-course within the left and right seeds of each ROI (except for the pgACC, comprising only one medial seed) was calculated and used as a regressor in a general linear model. In addition, white matter signal, cerebrospinal fluid signal, six motion parameters (three translations and three rotations), and the global signal were used as nuisance regressors. We have obtained white matter and cerebrospinal fluid masks using FSL’s FAST segmentation program. The resulting segmented images were then thresholded to ensure 80% tissue type probability. For each individual, the general linear model was analysed by using the FMRI Expert Analysis Tool, version 5.4, part of FMRIB’s Software Library (Smith et al., 2004). The resulting parameter estimate maps were then analysed using higher level 1 sample *t*-tests for group averages and between-samples *t*-tests for group differences. Clusters were determined by Z > 2.3 voxel-wise thresholding and a family-wise error-corrected cluster significance threshold of P < 0.05 (Worsley, 2001). From the results, we report only those that met the further correction for number of ROIs examined that gave *P *< 0.016 (i.e. *P <* 0.05 Bonferroni corrected for the three networks of interest: nucleus accumbens, dmPFC and pgACC (Davidson et al., 2003)). The % BOLD signal change data was extracted from the regions of significant effect (Table S2) using the FSL tool Featquery (www.fmrib.ox.ac.uk/fsl) (Smith et al., 2004) and using a dimensional approach was correlated with depression severity (BDI) and anhedonia (SHAPS, FCPS and TEPS) using Pearson correlations.

## Results

Table 1 shows the demographics of the study population; there were no significant differences between the DS group and controls for age, gender and BMI. Differences were present for depression: BDI, and anhedonia: SHAPS, FCPS, TEPS.

### Main effects of stimuli on blood oxygen level-dependent responses

Table S2 reports the main effects, i.e. the brain regions that had RSFC with the seed regions (baseline) for the HC group only. Overall, the patterns of connectivity associated with each of the seed regions are consistent with RSFC experiments in previous studies (Bebko et al., 2015; Clasen et al., 2014; Cullen et al., 2014; Guo et al., 2015; Sheline et al., 2009, 2010). Table 2 provides a summary of brain regions where there was a significant difference in connectivity between the seeds and the whole brain in those with depression symptoms (DS) vs. no symptoms (HC). These data we extracted and used to examine correlations with depression and anhedonia symptoms.

**Table 2.** RSFC between seed regions and whole brain compared between DS and HC groups controlled for medication status and age.

Brain Region	MNI Coordinates			z-score	Cluster size	*P* value
	X	Y	Z			
	Increased connectivity in DS vs. HC					
R dmPFC seed						
Frontal Pole	−32	32	12	4.11	485	<.001
ACC/Paracingulate	−8	25	22	3.2	485	<.001
L dmPFC seed						
Postcentral gyrus	54	−14	42	3.9	297	0.008
L NAcc seed						
Precuneus	−14	−60	34	3.86	238	0.008
Precuneus	6	−60	38	3.21	238	0.008
pgACC seed						
Thalamus	−2	−4	−4	4.27	655	<.001
Putamen	−26	4	0	4.12	655	<.001
Caudate	−10	8	16	3.8	655	<.001
NAcc	6	6	−2	3.51	655	<.001
Planum Temporale	−60	−38	14	4.64	286	0.008
STG	−66	−24	12	3.92	286	0.008
	Decreased connectivity in DS vs. HC					
R dmPFC seed						
Cuneal cortex	−2	−82	26	4.09	328	0.002
Precuneus	−20	−78	24	2.88	328	0.002
Precuneus	8	−76	36	3.1	282	0.002
L dmPFC seed						
ITG/MTG	58	−22	−26	4.14	407	<0.001
LOC	40	−84	8	4.03	388	0.001
pgACC seed						
SFG/MFG	−22	16	44	3.88	385	0.001
Postcentral gyrus	−38	−22	60	3.98	269	0.013

All *p*-values Z > 2.3 voxel-wise thresholding and a family-wise error-corrected cluster significance threshold of *P* < 0.05, further Bonferroni corrected for number of ROIs gave *P* < 0.012 (i.e. *P <* 0.05 (Davidson et al., 2003). ACC: anterior cingulate cortex; dmPFC: dorsal medial prefrontal cortex; IFG: inferior frontal gyrus; IFG: inferior temporal gurus; LOC: lateral occipital cortex; MFG: medial frontal gyrus; NAcc: nucleus accumbens; pgACC: pregenual anterior cingulate cortex; SFG: superior frontal gyrus; STG: superior temporal gyrus.

#### RSFC and Anhedonia: TEPS-A

**Figure 1.**
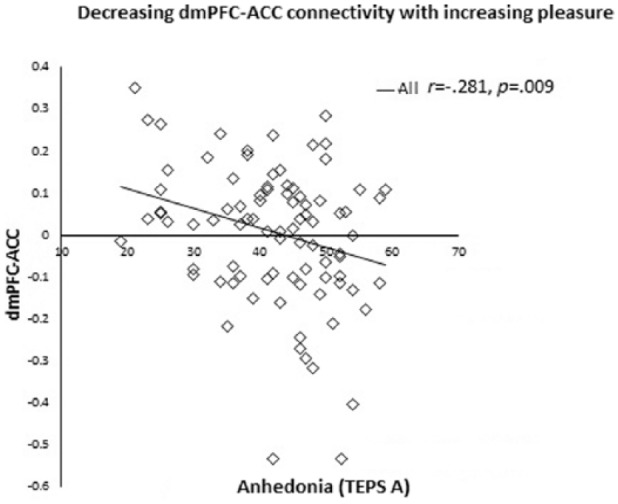
Negative correlation between RSFC of the right dmPFC seed and the ACC/paracingulate gyrus with TEPS anticipatory scale in all participants (*r *= –.281, *p *= .009, two-tailed).

There was a negative correlation between RSFC of the right dmPFC seed and the ACC/paracingulate gyrus with the TEPS anticipatory scale in all participants (*r *= –.281, *p *= .009) (Figure 1). Meaning that the higher the neural activity the lower the ability to anticipate pleasure. There was no significant correlations when separated into the DS and HC groups (p > 0.05).

**Figure 2.**
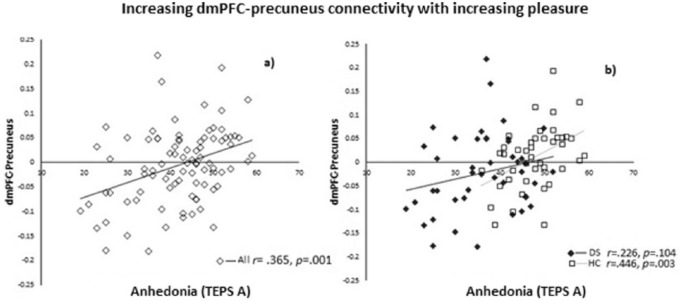
Positive correlation between RSFC of the right dmPFC seed and the left precuneus and TEPS anticipatory scale in (a) all participants (*r *= .365, *p *= .001, two-tailed), and (b) the HC group (*r *= –.446, *p *= .003, two-tailed) but not in the DS group (*r *= –.226, *p *= .104, two-tailed).

There was a positive correlation between RSFC of the right dmPFC seed and the left precuneus with the TEPS anticipatory scale in all participants (*r *= .365, *p *= .001, two-tailed). Meaning decreased connectivity correlated with anhedonia. The connectivity was also significant in the HC group (*r *= –.446, *p *= .003, two-tailed) but not in the DS group (p > 0.05) (Figure 2).

There were no significant correlations between the anhedonia measures FCPS and SHAPS and RSFC.

#### RSFC and depression severity: BDI

There was a positive correlation between RSFC of the right dmPFC seed and the frontal pole and BDI in all participants (*r *= .31, *p *= .004, two-tailed). Meaning increased connectivity correlated with increased depression severity. However, this connectivity did not remain significant when separated into the DS and HC groups alone (*p *> 0.05).

**Figure 3.**
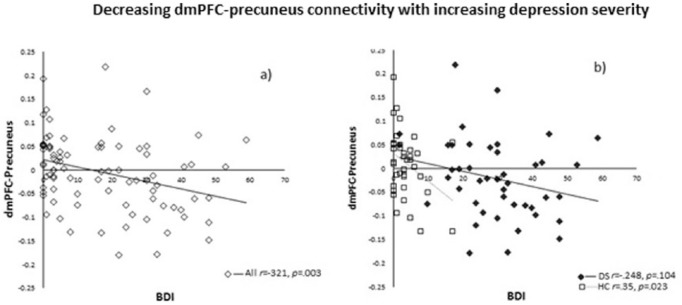
Negative correlation between RSFC of the right dmPFC seed and the left precuneus and BDI in (a) all participants (*r *= –.321, *p *= .003), and (b) the HC group (*r *= –.350, *p *= .023) but not the DS group (*r *= –.248, *p *= .104).

There was a negative correlation between RSFC of the right dmPFC seed and the left precuneus and BDI in all participants (*r *= –.321, *p *= .003, two-tailed). Meaning decreased connectivity correlated with increased depression severity. Also, this connectivity remained significant in the HC group (*r *= –.350, *p *= .023, two-tailed) but not in the DS group (p > 0.05) (Figure 3).

## Discussion

The aim of our study was to investigate how RSFC was related to a range of depression and anhedonia symptoms in adolescents. We selected key regions of interest shown to be dysfunctional in previous studies of RSFC in adults with depression.

We found anticipatory anhedonia related to increased dmPFC RSFC with the ACC/paracingulate gyrus, a part of the SN, across all participants. The dmPFC is a key node of the CEN network that is recruited by cognitively demanding tasks including working memory, attention and response inhibition (Seeley et al., 2007) (Garavan et al., 2002), and has been reported dysfunctional in individuals with depressive symptoms (Fonseka et al., 2016; Nixon et al., 2013; Sheline et al., 2010). Interestingly the ACC has been proposed as a bridge between attentional and emotional processing (Bush et al., 2000; Devinsky et al., 1995) that is also critical for self-regulation and adaptability (Zheng et al., 2017). Furthermore, the ACC has been highlighted as dysfunctional in depression during tasks and the resting state in adults (Sheline et al., 2010; Zheng et al., 2017) and adolescents (Pannekoek et al., 2014) and has been suggested as a predictor of treatment response in depression (Pizzagalli, 2011). Moreover, examining depressed adolescents (Gabbay et al., 2013) also found anhedonia severity correlated with increased pgACC RSFC with the caudate, although in the latter study anhedonia was measured by only two questions on the BDI and not with an anhedonia specific questionnaire as we have in our study. In our previous study examining young people with depression symptoms but no clinical diagnosis we also found increased pgACC RSFC correlating with anhedonia, consistent with this finding, albeit with a small sample (Rzepa and McCabe, 2016). Therefore, we suggest that the increased connectivity between the dmPFC and part of the ACC/paracingulate gyrus in this much larger study shows, for the first time, how decreased ability to specifically anticipate pleasure may serve as a mechanism for the emergence of anhedonia in young people.

We also found depression severity related to increased dmPFC RSFC with the frontal pole. The frontal pole has been found active during tasks that require cognitive effort or attention (Corbetta and Shulman, 2002) and is thought important for executive function and stimulus and goal-directed behaviour (Burgess et al., 2007; Orr et al., 2015). Our finding is somewhat consistent with that of Zhou et al. (2010), who reported increased RSFC associated with the lateral prefrontal cortices that also correlated with depressive episode duration and depression severity in depressed adult patients (mean age 38–40 years). The authors suggested that such dysfunctional network activity in areas involved in emotion, attention and memory may underpin negative bias, one of the main characteristics of depression. Therefore our results extend this idea by reporting on a younger sample, i.e. adolescents. Further, our result of dmPFC-frontal pole connectivity being related to depression severity and not anhedonia perhaps indicates a mechanism for altered negative rather than positive processing. Of course, it could also be related to an imbalance between positive and negative processing given our findings of altered dmPFC connectivity with other brain regions correlating with anhedonia.

Consistent with this, we also found decreased dmPFC RSFC with the precuneus, which correlated with anticipatory anhedonia *and* depression severity. The precuneus is a part of the DMN (Ralchle and Snyder, 2007), and thought to be involved in self-referential thoughts and rumination in depression (Burkhouse et al., 2017; Zhu et al., 2012). During rest or internally focused cognitions, activation of the CEN (including the dmPFC) decreases while DMN activation increases (Fox et al., 2005; Grady et al., 2010; Raichle et al., 2001). Furthermore, studies have shown that RSFC between the CEN and the DMN is altered in MDD (Hamilton et al., 2011; Manoliu et al., 2014a; Sheline et al., 2010), which has been suggested as being related to patients’ difficulties to disengage from negative thoughts (Manoliu et al., 2014a). A recent study found that those at high risk of depression due to a family history had decreased negative DMN-CEN connectivity (Posner et al., 2016), which the authors proposed as a possible indicator of risk for depression, although they did not report any relationship between network activity and depression symptoms in their study, whereas a recent study in healthy adolescents found decreased RSFC between the subgenual ACC and dmPFC, posterior cingulate, angular gyrus and middle temporal gyrus associated with higher depressive symptoms over time (Strikwerda-Brown et al., 2014). The authors suggested that reduced functional connectivity between key limbic and prefrontal regions may serve as a risk marker for greater depressive symptoms later in life. Interestingly, studies report causal influences of the SN in modulating the activity of the DMN and CEN (Bonnelle et al., 2012; Rilling et al., 2008; Sridharan et al., 2008); therefore, although speculative, the increased dmPFC- SN RSFC in this study might be causing the decreased RSFC we find with the DMN. Further studies are needed to test this directly. Our results are also consistent with the recent meta-analysis that describes a model of altered RSFC in mainly the fronto-limbic system and DMN (Zhong et al., 2016). Our results support this model in that these networks may contribute to emotional dysregulation, but we also extend the findings by showing how frontal cortex RSFC is related to anhedonia, whereas the DMN RSFC is related to both depression severity and anhedonia.

Of note, correlations between RSFC and symptoms were significant across the entire sample and in some cases in the HC group alone; none were significant in the DS group only. This suggests that the findings could be driven by the HC group (effect size *r* was also greater in the HC correlation alone than in the combined correlation) and that the relationship between brain FC and mood and pleasure are less aligned when symptoms become more severe. Furthermore the anhedonia questionnaire TEPS was designed to measure individual trait dispositions in both anticipatory and consummatory experiences of pleasure, which might explain how it was more easily mapped onto neural RSFC in our sample of adolescents with a range of symptoms compared with both the FCPS and the SHAPS, which measure state effects.

In conclusion, our findings show for the first time increased dmPFC RSFC with the SN and frontal pole, but also decreased dmPFC RSFC with the DMN correlating with depression severity and anhedonia in adolescents, lending further evidence to the importance of these networks as possible biomarkers for risk for depression.

## Supplemental Material

jop-2018-3438-File002 – Supplemental material for Anhedonia and depression severity dissociated by dmPFC resting-state functional connectivity in adolescents Click here for additional data file. Supplemental material, jop-2018-3438-File002 for Anhedonia and depression severity dissociated by dmPFC resting-state functional connectivity in adolescents by Ewelina Rzepa and Ciara McCabe in Journal of Psychopharmacology

## References

[bibr1-0269881118799935] Bebko G Bertocci M Chase H et al (2015) Decreased amygdala-insula resting state connectivity in behaviorally and emotionally dysregulated youth. Psychiatry Res 231: 77–86. 25433424 10.1016/j.pscychresns.2014.10.015 PMC4272653

[bibr2-0269881118799935] Bluhm R Williamson P Lanius R et al (2009) Resting state default-mode network connectivity in early depression using a seed region-of-interest analysis: Decreased connectivity with caudate nucleus. Psychiatry Clin Neurosci 63: 754–761. 20021629 10.1111/j.1440-1819.2009.02030.x

[bibr3-0269881118799935] Bonnelle V Ham TE Leech R et al (2012) Salience network integrity predicts default mode network function after traumatic brain injury. Proc Natl Acad Sci USA 109: 4690–4695. 22393019 10.1073/pnas.1113455109 PMC3311356

[bibr4-0269881118799935] Bressler SL Menon V (2010) Large-scale brain networks in cognition: Emerging methods and principles. Trends Cogn Sci 14: 277–290. 20493761 10.1016/j.tics.2010.04.004

[bibr5-0269881118799935] Burgess PW Gilbert SJ Dumontheil I (2007) Function and localization within rostral prefrontal cortex (area 10). Philos Trans R Soc B Biol Sci 362: 887–899. 10.1098/rstb.2007.2095 PMC2430004 17403644

[bibr6-0269881118799935] Burkhouse KL Jacobs RH Peters AT et al (2017) Neural correlates of rumination in adolescents with remitted major depressive disorder and healthy controls. Cogn Affect Behav Neurosci 17: 394–405. 27921216 10.3758/s13415-016-0486-4 PMC5366093

[bibr7-0269881118799935] Bush G Luu P Posner MI (2000) Cognitive and emotional influences in anterior cingulate cortex. Trends Cogn Sci 4: 215–222. 10827444 10.1016/s1364-6613(00)01483-2

[bibr8-0269881118799935] Clasen PC Beevers CG Mumford JA et al (2014) Cognitive control network connectivity in adolescent women with and without a parental history of depression. Dev Cogn Neurosci 7: 13–22. 24270043 10.1016/j.dcn.2013.10.008 PMC4209722

[bibr9-0269881118799935] Corbetta M Shulman GL (2002) Control of goal-directed and stimulus-driven attention in the brain. Nat Rev Neurosci 3: 201–215. 11994752 10.1038/nrn755

[bibr10-0269881118799935] Cowdrey FA Filippini N Park RJ et al (2012) Increased resting state functional connectivity in the default mode network in recovered anorexia nervosa. Hum Brain Mapp 35: 483–491. 23033154 10.1002/hbm.22202 PMC6869597

[bibr11-0269881118799935] Cullen KR Westlund MK Klimes-dougan B et al (2014) Abnormal amygdala resting-state functional connectivity in adolescent depression. JAMA Psychiatry 71: 1138–1147. 25133665 10.1001/jamapsychiatry.2014.1087 PMC4378862

[bibr12-0269881118799935] Davidson RJ Irwin W Anderle MJ et al (2003) The neural substrates of affective processing in depressed patients treated with venlafaxine. Am J Psychiatry 160: 64–75. 12505803 10.1176/appi.ajp.160.1.64

[bibr13-0269881118799935] Devinsky O Morrell MJ Vogt BA (1995) Contributions of anterior cingulate cortex to behaviour. Brain 118: 279–306. 7895011 10.1093/brain/118.1.279

[bibr14-0269881118799935] Fawcett J Clark DC Scheftner WA et al (1983) Assessing anhedonia in psychiatric patients. Arch Gen Psychiatry 40: 79–84. 6849623 10.1001/archpsyc.1983.01790010081010

[bibr15-0269881118799935] First MB Spitzer RL Gibbon M et al (1997) Structured Clinical Interview for DSM-IV Axis I Disorders: Clinical Version. Washington, DC: American Psychiatric Press.

[bibr16-0269881118799935] Fonseka BA Jaworska N Courtright A et al (2016) Cortical thickness and emotion processing in young adults with mild to moderate depression: A preliminary study. BMC Psychiatry 16: 38. 26911621 10.1186/s12888-016-0750-8 PMC4765096

[bibr17-0269881118799935] Fox MD Snyder AZ Vincent JL et al (2005) The human brain is intrinsically organized into dynamic, anticorrelated functional networks. Proc Natl Acad Sci USA 102: 9673–9678. 15976020 10.1073/pnas.0504136102 PMC1157105

[bibr18-0269881118799935] Gabbay V Ely BA Li QY et al (2013) Striatum-Based circuitry of adolescent depression and anhedonia. J Am Acad Child Adolesc Psychiatry 52: 628–641. 23702452 10.1016/j.jaac.2013.04.003 PMC3762469

[bibr19-0269881118799935] Garavan H Ross TJ Murphy K et al (2002) Dissociable executive functions in the dynamic control of behavior: Inhibition, error detection, and correction. Neuroimage 17: 1820–1829. 12498755 10.1006/nimg.2002.1326

[bibr20-0269881118799935] Grady CL Protzner AB Kovacevic N et al (2010) A multivariate analysis of age-related differences in default mode and task-positive networks across multiple cognitive domains. Cereb Cortex 20: 1432–1447. 19789183 10.1093/cercor/bhp207 PMC3181214

[bibr21-0269881118799935] Greicius MD Flores BH Menon V et al (2007) Resting-state functional connectivity in major depression: Abnormally increased contributions from subgenual cingulate cortex and thalamus. Biol Psychiatry 62: 429–437. 17210143 10.1016/j.biopsych.2006.09.020 PMC2001244

[bibr22-0269881118799935] Guo W Liu F Chen J et al (2015) Resting-state cerebellar-cerebral networks are differently affected in first-episode, drug-naive schizophrenia patients and unaffected siblings. Sci Rep 5: 17275. 26608842 10.1038/srep17275 PMC4660304

[bibr23-0269881118799935] Hamilton JP Furman DJ Chang C et al (2011) Default-mode and task-positive network activity in major depressive disorder: Implications for adaptive and maladaptive rumination. Biol Psychiatry 70: 327–333. 21459364 10.1016/j.biopsych.2011.02.003 PMC3144981

[bibr24-0269881118799935] Horn DI Yu C Steiner J et al (2010) Glutamatergic and resting-state functional connectivity correlates of severity in major depression – the role of pregenual anterior cingulate cortex and anterior insula. Front Syst Neurosci 4: pii: 33. 10.3389/fnsys.2010.00033 PMC2914530 20700385

[bibr25-0269881118799935] Insel T Cuthbert B Garvey M et al (2010) Research domain criteria (RDoC): Toward a new classification framework for research on mental disorders. Am Psychiatric Assoc 167: 748–751. 10.1176/appi.ajp.2010.09091379 20595427

[bibr26-0269881118799935] Jenkinson M Smith S (2002) A global optimisation method for robust affine registration of brain images. Med Image Anal 5: 143–156. 10.1016/s1361-8415(01)00036-6 11516708

[bibr27-0269881118799935] Kennedy DN Lange N Makris N et al (1998) Gyri of the human neocortex: An MRI-based analysis of volume and variance. Cereb Cortex 8: 372–384. 9651132 10.1093/cercor/8.4.372

[bibr28-0269881118799935] Manoliu A Meng C Brandl F et al (2014a) Insular dysfunction within the salience network is associated with severity of symptoms and aberrant inter-network connectivity in major depressive disorder. Front Hum Neurosci 7: 930. 24478665 10.3389/fnhum.2013.00930 PMC3896989

[bibr29-0269881118799935] Manoliu A Meng C Brandl F et al (2014b) Insular dysfunction within the salience network is associated with severity of symptoms and aberrant inter-network connectivity in major depressive disorder. Front Hum Neurosci 7: 930. 24478665 10.3389/fnhum.2013.00930 PMC3896989

[bibr30-0269881118799935] McCabe C Mishor Z (2011) Antidepressant medications reduce subcortical-cortical resting-state functional connectivity in healthy volunteers. Neuroimage 57: 1317–1323. 21640839 10.1016/j.neuroimage.2011.05.051 PMC3141109

[bibr31-0269881118799935] McCabe C Mishor Z Filippini N et al (2011) SSRI administration reduces resting state functional connectivity in dorso-medial prefrontal cortex. Mol Psychiatry 16: 592–594. 21263442 10.1038/mp.2010.138

[bibr32-0269881118799935] Nixon NL Liddle PF Worwood G et al (2013) Prefrontal cortex function in remitted major depressive disorder. Psychol Med 43: 1219–1230. 23020994 10.1017/S0033291712002164

[bibr33-0269881118799935] Northoff G (2016) How do resting state changes in depression translate into psychopathological symptoms? From ‘spatiotemporal correspondence’ to ‘spatiotemporal psychopathology’. Cur Opin Psychiatry 29: 18–24. 10.1097/YCO.0000000000000222 26651006

[bibr34-0269881118799935] Oathes DJ Patenaude B Schatzberg AF et al (2015) Neurobiological signatures of anxiety and depression in resting-state functional magnetic resonance imaging. Biol Psychiatry 77: 385–393. 25444162 10.1016/j.biopsych.2014.08.006 PMC4297561

[bibr35-0269881118799935] Orr JM Smolker HR Banich MT (2015) Organization of the human frontal pole revealed by large-scale DTI-based connectivity: Implications for control of behavior. PLoS One 10: e0124797. 25945925 10.1371/journal.pone.0124797 PMC4422440

[bibr36-0269881118799935] Pannekoek JN van der Werff SJ Meens PH et al (2014) Aberrant resting-state functional connectivity in limbic and salience networks in treatment – naive clinically depressed adolescents. J Child Psychol Psychiatry 55: 1317–1327. 24828372 10.1111/jcpp.12266

[bibr37-0269881118799935] Patriat R Molloy EK Meier TB et al (2013) The effect of resting condition on resting-state fMRI reliability and consistency: A comparison between resting with eyes open, closed, and fixated. Neuroimage 78: 463–473. 23597935 10.1016/j.neuroimage.2013.04.013 PMC4003890

[bibr38-0269881118799935] Pizzagalli DA (2011). Frontocingulate dysfunction in depression: Toward biomarkers of treatment response. Neuropsychopharmacology 36: 183–206. 20861828 10.1038/npp.2010.166 PMC3036952

[bibr39-0269881118799935] Posner J Cha J Wang ZS et al (2016) Increased default mode network connectivity in individuals at high familial risk for depression. Neuropsychopharmacology 41: 1759–1767. 26593265 10.1038/npp.2015.342 PMC4869043

[bibr40-0269881118799935] Raichle ME Macleod AM Snyder AZ et al (2001) A default mode of brain function. Proc Natl Acad Sci USA 98: 676–682. 11209064 10.1073/pnas.98.2.676 PMC14647

[bibr41-0269881118799935] Ralchle ME Snyder AZ (2007) A default mode of brain function: A brief history of an evolving idea. Neuroimage 37: 1083–1090. 17719799 10.1016/j.neuroimage.2007.02.041

[bibr42-0269881118799935] Ramasubbu R Konduru N Cortese F et al (2014) Reduced intrinsic connectivity of amygdala in adults with major depressive disorder. Front Psychiatry 5: 17. 24600410 10.3389/fpsyt.2014.00017 PMC3928548

[bibr43-0269881118799935] Rilling JK Dagenais JE Goldsmith DR et al (2008) Social cognitive neural networks during in-group and out-group interactions. Neuroimage 41: 1447–1461. 18486491 10.1016/j.neuroimage.2008.03.044

[bibr44-0269881118799935] Rzepa E Fisk J McCabe C (2016a) Blunted neural response to anticipation, effort and consummation of reward and aversion in adolescents with depression symptomatology. J Psychopharmacol 31: 303–311. 10.1177/0269881116681416 28093022

[bibr45-0269881118799935] Rzepa E McCabe C (2016) Decreased anticipated pleasure correlates with increased salience network resting state functional connectivity in adolescents with depressive symptomatology. J Psychiatr Res 82: 40–47. 27459031 10.1016/j.jpsychires.2016.07.013 PMC5036507

[bibr46-0269881118799935] Rzepa E Tudge L McCabe C (2016b) The CB1 neutral antagonist tetrahydrocannabivarin reduces default mode network and increases executive control network resting state functional connectivity in healthy volunteers. Int J Neuropsychopharmacol 19: pyv092. 10.1093/ijnp/pyv092 PMC4772823 26362774

[bibr47-0269881118799935] Saluja G Iachan R Scheidt PC et al (2004) Prevalence of and risk factors for depressive symptoms among young adolescents. Arch Pediatr Adolesc Med 158: 760–765. 15289248 10.1001/archpedi.158.8.760

[bibr48-0269881118799935] Sawyer SM Azzopardi PS Wickremarathne D et al (2018) The age of adolescence. Lancet Child Adolesc Health 2: 223–228. 30169257 10.1016/S2352-4642(18)30022-1

[bibr49-0269881118799935] Seeley WW Menon V Schatzberg AF et al (2007) Dissociable intrinsic connectivity networks for salience processing and executive control. J Neurosci 27: 2349–2356. 17329432 10.1523/JNEUROSCI.5587-06.2007 PMC2680293

[bibr50-0269881118799935] Sheline YI Barch DM Price JL et al (2009) The default mode network and self-referential processes in depression. Proc Natl Acad Sci USA 106: 1942–1947. 19171889 10.1073/pnas.0812686106 PMC2631078

[bibr51-0269881118799935] Sheline YI Price JL Yan Z et al (2010) Resting-state functional MRI in depression unmasks increased connectivity between networks via the dorsal nexus. Proc Natl Acad Sci USA 107: 11020–11025. 20534464 10.1073/pnas.1000446107 PMC2890754

[bibr52-0269881118799935] Smith SM (2002) Fast robust automated brain extraction. Hum Brain Mapp 17: 143–155. 12391568 10.1002/hbm.10062 PMC6871816

[bibr53-0269881118799935] Smith SM Jenkinson M Woolrich MW et al (2004) Advances in functional and structural MR image analysis and implementation as FSL. Neuroimage 23(Suppl 1): S208–S219. 15501092 10.1016/j.neuroimage.2004.07.051

[bibr54-0269881118799935] Snaith RP Hamilton M Morley S et al (1995) A scale for the assessment of hedonic tone the Snaith–Hamilton pleasure scale. Br J Psychiatry 167: 99–103. 7551619 10.1192/bjp.167.1.99

[bibr55-0269881118799935] Sridharan D Levitin DJ Menon V (2008) A critical role for the right fronto-insular cortex in switching between central-executive and default-mode networks. Proc Natl Acad Sci USA 105: 12569–12574. 18723676 10.1073/pnas.0800005105 PMC2527952

[bibr56-0269881118799935] Strikwerda-Brown C Davey CG Whittle S et al (2014) Mapping the relationship between subgenual cingulate cortex functional connectivity and depressive symptoms across adolescence. Soc Cogn Affect Neurosci 10: 961–968. 25416726 10.1093/scan/nsu143 PMC4483565

[bibr57-0269881118799935] Tahmasian M Knight DC Manoliu A et al (2013) Aberrant intrinsic connectivity of hippocampus and amygdala overlap in the fronto-insular and dorsomedial-prefrontal cortex in major depressive disorder. Front Hum Neurosci 7: 639. 24101900 10.3389/fnhum.2013.00639 PMC3787329

[bibr58-0269881118799935] van Tol MJ Veer IM van der Wee NJ et al (2013) Whole-brain functional connectivity during emotional word classification in medication-free major depressive disorder: Abnormal salience circuitry and relations to positive emotionality. Neuroimage Clin 2: 790–796. 24179829 10.1016/j.nicl.2013.05.012 PMC3777780

[bibr59-0269881118799935] Worsley K (2001) Statistical analysis of activation images. In: Jezzard P Matthews PM Smith SM (eds) Functional MRI: An Introduction to Methods, vol. 14 Oxford: Oxford University Press, pp. 251–270.

[bibr60-0269881118799935] Ye T Peng J Nie BB et al (2012) Altered functional connectivity of the dorsolateral prefrontal cortex in first-episode patients with major depressive disorder. Eur J Radiol 81: 4035–4040. 22939367 10.1016/j.ejrad.2011.04.058

[bibr61-0269881118799935] Zheng H Li F Bo Q et al (2017) The dynamic characteristics of the anterior cingulate cortex in resting-state fMRI of patients with depression. J Affect Disord 227: 391–397. 29154155 10.1016/j.jad.2017.11.026

[bibr62-0269881118799935] Zhong X Pu W Yao S (2016) Functional alterations of fronto-limbic circuit and default mode network systems in first-episode, drug-naive patients with major depressive disorder: A meta-analysis of resting-state fMRI data. J Affect Disord 206: 280–286. 27639862 10.1016/j.jad.2016.09.005

[bibr63-0269881118799935] Zhou Y Yu C Zheng H et al (2010) Increased neural resources recruitment in the intrinsic organization in major depression. J Affect Disord 121: 220–230. 19541369 10.1016/j.jad.2009.05.029

[bibr64-0269881118799935] Zhu X Wang X Xiao J et al (2012) Evidence of a dissociation pattern in resting-state default mode network connectivity in first-episode, treatment-naive major depression patients. Biol Psychiatry 71: 611–617. 22177602 10.1016/j.biopsych.2011.10.035

