# Supplementary material for: Anhedonia and depression severity dissociated by dmPFC resting-state functional connectivity in adolescents
Source: J Psychopharmacol. 2018 Sep 27;32(10):1067–74. doi: 10.1177/0269881118799935 (PMC6380625; doi:10.1177/0269881118799935)
Supplement: jop-2018-3438-File002 – Supplemental material for Anhedonia and depression severity dissociated by dmPFC resting-state functional connectivity in adolescents [file jop-2018-3438-File002.pdf]

Supplementary data

Anhedonia and depression severity dissociated by dmPFC Resting-State Functional Connectivity in Adolescents.

Running title:

RSFC and depression symptoms in adolescence

Category:

Regular research article

Ewelina Rzepa PhD, Ciara McCabe* PhD.

School of Psychology and Clinical Language Sciences, University of Reading, UK.

* Corresponding author:

Dr Ciara McCabe

Associate Professor of Neuroscience,

School of Psychology and Clinical Language Sciences,

University of Reading,

Reading RG6 6AL,

Tel: +44 118 378 5450

c.mccabe@reading.ac.uk

Table S1: Medication status of participants with MDD diagnosis.

Participant ID	Medicated during study	History of medications
1	Citalopram 10mg	Sertraline
2	-	Bupropion, Fluoxetine, Sertraline, Mirtazapine
3	Fluoxetine 50mg	-
4	Sertraline 100mg	
5	-	Fluoxetine 20-60mg, Sertraline 50mg
6	Sertraline 100mg	Citalopram
7	Sertraline	-
8	-	Fluoxetine 20mg
9	Citalopram 20mg	-
10	Citalopram 40mg	-
11	-	Citalopram 20mg
12	Citalopram 30mg	-
13	Fluoxetine 40mg	-
14	Sertraline 10mg	-
15	Fluoxetine 60mg	-
16	-	Fluoxetine 20mg
17	-	Citalopram 20mg
18	Paroxetine 20mg	-
19	-	Citalopram 20mg
20	-	Fluoxetine 20mg
21	Citalopram 40mg	-
22	Sertraline 50mg	-

Table S2. Functional connectivity between seeds and whole brain in healthy controls.

Brain regions	MNI coordinates			
	X	Y	Z	z-score
Left dmPFC				
Frontal pole	-24	36	28	10.6
Frontal Pole	-24	46	24	7.79
	32	42	34	6.28
ACC/paracing	-10	36	26	6.39
Frontal pole	28	30	36	4.68
Frontal Operculum Cortex/insula	-40	20	2	4.13
Right dmPFC				
Frontal Pole	18	34	30	10.7
ACC/Paracing	2	44	28	6.5
Superior Frontal Gyrus	12	36	40	6.61
Middle Temporal Gyrus	64	0	-24	5.13
Temporal Pole	54	4	-38	4.26

LOC	8	-58	8	4.24
Cuneal cortex/Precuneus	4	-70	24	4.43
Precuenus	6	-54	18	4.03
L NAcc				
NAcc	-8	10	-6	13.6
OFC	20	8	-18	5.58
Paracingulate Gyrus	-2	54	4	5.58
OFC	-18	6	-24	5.36
Precuenus/PCC	-4	-52	10	4.21
Precuenus/PCC	12	-52	4	4.09
R NAcc				
NAcc	10	10	-8	13.7
Paracingulate Gyrus	16	2	-28	5.04
Frontal Medial cortex	-6	32	-18	4.76
Frontal Pole	22	38	-22	4.72
Precuenus/PCC	4	-54	6	4.03
PCC	8	-46	4	3.76
PCC	-4	-48	32	2.85
Frontal Pole	-44	52	0	3.61
Parahippocampal gyrus	-28	-38	-4	2.76
Hippocampus	-22	-32	-10	2.33
pgACC				
pgACC	0	32	0	8.8
ACC/Paracingulate	-6	46	4	8.37
Frontal Pole	-10	56	14	6.79
Middle Temporal Gyrus	64	-4	-16	6.5
OFC/Insula	30	14	-18	5.65
LOC	-52	-64	36	5.06
LOC	50	-66	40	3.24
Parahippocampal gyrus	26	-22	-18	6.13

OFC- orbitofrontal cortex; PCC- posterior cingulate cortex; ACC- anterior cingulate cortex; pgACC-pregenual anterior cingulate cortex, LOC-Lateral Occipital Cortex

Table S3: RSFC between seed regions and whole brain compared between DS and HC groups controlled for medication status and age.

Brain Region	MNI Coordinates			z-score	Cluster size	P value
	X	Y	Z			

		Increased connectivity in DS vs. HC				
R dmPFC seed						
Frontal Pole	-32	32	12	4.11	485	<.001
ACC/Paracingulate	-8	25	22	3.2	485	<.001
L dmPFC seed						
Postcentral gyrus	54	-14	42	3.9	297	0.008
L NAcc seed						
Precuneus	-14	-60	34	3.86	238	0.008
Precuneus	6	-60	38	3.21	238	0.008
pgACC seed						
Thalamus	-2	-4	-4	4.27	655	<.001
Putamen	-26	4	0	4.12	655	<.001
Caudate	-10	8	16	3.8	655	<.001
NAcc	6	6	-2	3.51	655	<.001
Planum Temporale	-60	-38	14	4.64	286	0.008
STG	-66	-24	12	3.92	286	0.008
		Decreased connectivity in DS vs. HC				
R dmPFC seed						
Cuneal cortex	-2	-82	26	4.09	328	0.002
Precuneus	-20	-78	24	2.88	328	0.002
Precuneus	8	-76	36	3.1	282	0.002
L dmPFC seed						
ITG/MTG	58	-22	-26	4.14	407	<0.001
LOC	40	-84	8	4.03	388	0.001
pgACC seed						
SFG/MFG	-22	16	44	3.88	385	0.001
Postcentral gyrus	-38	-22	60	3.98	269	0.013

All *p*-values Z > 2.3 voxel-wise thresholding and a family-wise error-corrected cluster significance threshold of *P* < 0.05, further Bonferroni corrected for number of ROIs gave *P* < 0.012 (i.e, *P* < 0.05 (Davidson et al., 2003). pgACC- pregenual anterior cingulate cortex, ACC-anterior cingulate cortex, dmPFC- dorsal medial prefrontal cortex, LOC- lateral Occipital Cortex, NAcc- nucleus accumbens, STG- Superior Temporal gyrus, IFG- Inferior temporal gurus, SFG- Superior Frontal Gyrus, MFG- Medial frontal gyrus, IFG- inferior frontal gyrus, ACC-anterior cingulate cortex, dmPFC- dorsal medial prefrontal cortex, LOC- lateral Occipital Cortex, NAcc-nucleus accumbens, MFG- Medial frontal gyrus, IFG- inferior frontal gyrus.
